# Cancer Cachexia: Its Mechanism and Clinical Significance

**DOI:** 10.3390/ijms22168491

**Published:** 2021-08-06

**Authors:** Hiroki Nishikawa, Masahiro Goto, Shinya Fukunishi, Akira Asai, Shuhei Nishiguchi, Kazuhide Higuchi

**Affiliations:** 1 The Second Department of Internal Medicine, Osaka Medical and Pharmaceutical University, Takatsuki 569-8686, Japan; masahiro.goto@ompu.ac.jp (M.G.); in2104@osaka-med.ac.jp (S.F.); in2108@osaka-med.ac.jp (A.A.); kazuhide.higuchi@ompu.ac.jp (K.H.); 2 The Premier Departmental Research of Medicine, Osaka Medical and Pharmaceutical University, Takatsuki 569-8686, Japan; 3 Kano General Hospital, Osaka 531-0041, Japan; nishiguchi@heartfull.or.jp

**Keywords:** cachexia, advanced cancer, weight loss, mechanism, multidisciplinary intervention

## Abstract

The term “cachexia” is derived from the Greek words kakos (bad) and hexis (habit). Cachexia is a malnutrition associated with chronic diseases such as cancer, chronic heart failure, chronic renal failure, and autoimmune diseases, and is characterized by decreased skeletal muscle mass. Cancer cachexia is quite common in patients with advanced cancer. Weight loss is also a characteristic symptom of cancer cachexia, along with decreased skeletal muscle mass. As nutritional supplementation alone cannot improve cachexia, cytokines and tumor-derived substances have been attracting attention as its relevant factors. Cancer cachexia can be also associated with reduced chemotherapeutic effects, increased side effects and treatment interruptions, and even poorer survival. In 2011, a consensus definition of cachexia has been proposed, and the number of relevant research reports has increased significantly. However, the pathogenesis of cachexia is not fully understood, and there are currently few regulatory-approved standard treatments for cachexia. The main reason for this is that multiple etiologies are involved in the development of cachexia. In this review, we will outline the current status of cachexia, the mechanisms of which have been elucidated in recent years, especially from the perspective of advanced cancer.

## 1. Introduction

Cachexia is a term that has been used for a long time to describe a state of wasting due to poor nutrition [1]. The term “cachexia” is derived from the Greek words kakos (bad) and hexis (habit) [2]. Cachexia is a malnutrition associated with chronic diseases such as cancer, chronic heart failure, chronic renal failure, and autoimmune diseases, and is characterized by decreased skeletal muscle mass. Weight loss is also a characteristic symptom of cancer cachexia, along with decreased skeletal muscle mass [3,4]. From a nutritional standpoint, the factors associated with this condition were thought to be anorexia and increased energy expenditure [5]. However, as nutritional supplementation alone cannot improve cachexia, cytokines and tumor-derived substances have been attracting attention as its relevant factors since around the late 1990s [6,7]. Cancer cachexia is now considered to be a condition caused by metabolic, immunological, and neurological abnormalities rather than mere nutritional abnormalities [6,7].

Decreased skeletal muscle mass, muscle strength and function have been reported to have adverse effects on QOL and life expectancy, and have recently become widely known as sarcopenia [2,8]. With the aging population in developed countries, countermeasures against aging-related sarcopenia are attracting attention [9,10,11]. On the other hand, cachexia, which is secondary to sarcopenia caused by chronic diseases, remains less recognized among medical professionals compared to sarcopenia, although there are many opportunities to encounter it in daily practice [2,12,13]. There are many cases of undiagnosed cachexia in which the nutritional status deteriorates, resulting in further loss of skeletal muscle mass and physical functional decline. In 2011, a consensus definition of cachexia has been proposed, and the number of relevant research reports has increased significantly [14]. However, the pathogenesis of cachexia is not fully understood, and there are currently few regulatory-approved standard treatments for cachexia [15]. The main reason for this is that multiple etiologies are involved in the development of cachexia. For a long time, cachexia had been understood as an inevitable condition associated with the progression of underlying diseases and was not subject to treatment or research, but various subsequent studies have gradually revealed that various molecular mechanisms in skeletal muscle, adipose tissue, digestive system, central nervous system, and immune system are involved in the development of cachexia [16]. Particularly, in cancer cachexia, substances secreted by the tumor and tumor-induced immune responses and metabolic changes have been suggested to be deeply involved in its pathogenesis [16]. Cancer cachexia is quite common in patients with advanced cancer [17,18]. Cancer cachexia can also be associated with reduced chemotherapeutic effects, increased side effects and treatment interruptions, and even poorer survival [19,20]. Weight loss in advanced cancer patients worsens the prognosis and often requires aggressive therapeutic intervention [19,21]. Thus, cancer cachexia is a condition that should not be overlooked.

In this review, we will outline the current status of cachexia, the mechanisms of which have been elucidated in recent years, especially from the perspective of advanced cancer.

## 2. Definition and Diagnostic Criteria for Cachexia

### 2.1. Definition and Diagnostic Criteria

At a consensus meeting held in Washington, D.C., in late 2006, Evans and other experts from Europe and the USA stated, “Cachexia is a syndrome with complex metabolic abnormalities caused by an underlying disease and characterized by loss of skeletal muscle mass with or without loss of fat mass. Cachexia-related clinical manifestations include weight loss in adults and impaired growth in children. Anorexia, inflammation, insulin resistance, and muscle proteolysis are frequently observed. Cachexia is a condition distinct from starvation, aging-related loss of muscle mass, depression, malabsorption, and hyperthyroidism” [12]. Although starvation is also accompanied by weight loss, cancer cachexia differs from starvation in that the balance between skeletal muscle synthesis and breakdown is impaired, and resting energy expenditure (REE) is also increased [22]. In other words, cachexia is “eating and losing weight”, while starvation is “not being able to eat and losing weight”. The similarities and differences between cachexia and starvation are shown in Table 1. In 2011, Fearon et al. defined cachexia as (1) weight loss of 5% or more within 6 months, (2) weight loss of 2% or more in patients with a body mass index (BMI) <20 kg/m^2^, or (3) weight loss of 2% or more in patients with sarcopenia [14]. These definitions became the basis for subsequent cachexia studies, and the number of relevant reported studies increased significantly. A 2017 classification of malnutrition from the European Society of Clinical Nutrition and Metabolism (ESPEN) synonymous cachexia with “chronic disease-associated malnutrition with inflammation”, and it has been shown that the pathology is different from starvation and malabsorption without inflammation [23]. In 2018, a new criterion for cachexia specific to advanced cancer has been proposed [24]. This criterion is called cachexia staging score (CSS) and includes weight loss in 6 months (0–3 points), SARC-F (a screening tool for sarcopenia [25], 0–3 points), ECOG-PS (0–2 points), appetite loss (0–2 points), and abnormal biochemistry (0–2 points), and thus it consists of five items (total of 12 points) [24]. When classified into the four groups according to CSS (non-cachexia (0–2 points), pre-cachexia (3–4 points), cachexia (5–8 points), and refractory cachexia (9–12 points)), subjects with more advanced cachexia stages had lower skeletal muscle index, higher prevalence of sarcopenia, more severe symptom burden, poorer QOL, and shorter length of survival [24]. In addition, in 2021, the European Society of Medical Oncology (ESMO) issued clinical practice guidelines for patients with cancer cachexia [26]. It recommends defining cachexia as “disease-related malnutrition based on the Global leadership Initiative in Malnutrition (GLIM) definition [27] and the presence of systemic inflammation” [26].

### 2.2. Difficulties for the Use of Cachexia in Daily Clinical Practice

As mentioned above, several diagnostic criteria for cachexia have been published, but they are not widely used in daily practice. There are several reasons why it is difficult to develop diagnostic criteria for cachexia: (1) due to the effects of ascites and other factors, it is not easy to accurately assess the loss of skeletal muscle mass, which is the main cause of cachexia (in particular, the bioelectrical impedance analysis (BIA) method of muscle mass assessment overestimates muscle mass in patients with massive ascites or marked edema [28]); (2) weight and BMI, which have been classically used to diagnose cachexia, are not suitable for assessment methods in sarcopenic obese patients (sarcopenic patients with high BMI) and patients with severe edema because weight is rather increased due to increased fat mass and body water while skeletal muscle mass is decreased [29]; (3) standard values for skeletal muscle mass and BMI differ between Western countries and Asian countries; and (4) the etiology of cachexia is complex, and the phenotype and the progression rate of cachexia can vary depending on the underlying disease [30].

## 3. Three Stages in Cancer Cachexia

### 3.1. Pre-Cachexia, Cachexia, and Refractory Cachexia

As described above, a definition and diagnostic criteria for cancer cachexia was published in 2011 [14]. It classified cancer cachexia into the three stages: pre-cachexia, cachexia, and refractory cachexia, and recommended early intervention for pre-cachexia [14]. The concept of pre-cachexia has been proposed as a stage prior to the onset of cachexia, which is an obvious state of malnutrition [14,31]. Pre-cachexia is a condition associated with mild weight loss, inflammatory response, and anorexia. Although (1) weight loss of 5% or less, (2) anorexia, and (3) metabolic abnormalities have been proposed as diagnostic criteria, sufficient consensus has not been reached, and clinical features have only been described [2,14]. Pre-cachexia is an important concept proposed to prevent the progression of malnutrition through early nutritional care for patients at high risk for cachexia. Prevention of weight loss by early intervention may also lead to continuation of cancer treatment and favorable prognosis [14,19,21,32]. Martin et al. proposed a prognostic model based on weight loss in patients with advanced cancer. They reported that when the degree of weight loss was classified into the five grades (0–4), the median survival time was 21, 15, 11, 8, and 4 months, respectively, indicating the close correlation between the severity of weight loss and prognosis [21].

### 3.2. Treatment Strategies

**Figure 1:**
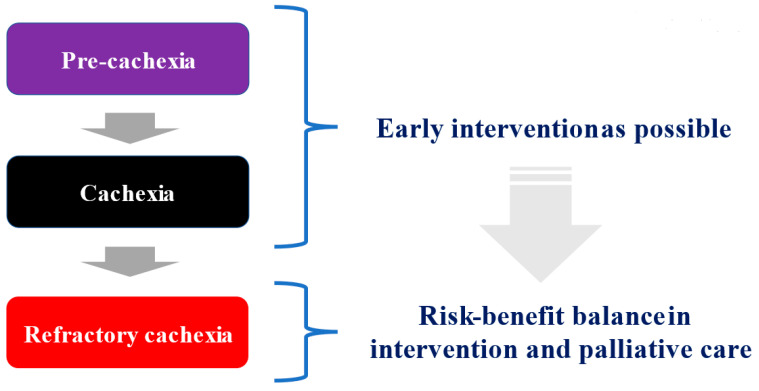
Three stages for cancer cachexia.

The therapeutic strategy is to address coexisting treatable factors. For example, appropriate treatment of nutrition impact symptoms (NIS) such as chemotherapy-induced oral mucosal damage, diarrhea, and nausea and vomiting, which affect oral intake and result in secondary starvation, is the first step [19,32]. However, as it is difficult to accurately identify the early stage of cachexia when the findings and signs of malnutrition are not obvious, useful biomarkers have been investigated [33]. Refractory cachexia is defined as (1) symptoms of cachexia plus increased catabolism and resistance to anticancer therapy, (2) ECOG-PS 3 or 4, and (3) a life expectancy of less than 3 months [14]. Although there is no clear evidence on when to end intervention, excessive intervention in patients with refractory cachexia may increase patient burden. Therefore, the risk–benefit ratio must be carefully evaluated while balancing palliative care in patients with refractory cachexia [34] (Figure 1).

## 4. Anabolic Resistance and Mechanism for Cachexia

### 4.1. Clinical Features for Anabolic Resistance

Anabolic resistance refers to a state of resistance to anabolism in which protein synthesis in muscle tissue fails to be done normally due to factors such as surgery, trauma, various chronic wasting diseases, aging, lack of exercise, and corticosteroid administration [35,36]. In many cases, patients with cachexia have a high level of anabolic resistance due to chronic inflammation from the underlying disease and additional factors such as aging and immobility. As the underlying disease worsens, the inflammatory response and metabolic abnormalities become more severe, and thus anabolic resistance becomes more advanced [35,36]. As mentioned earlier, systemic inflammation is one of the main pathophysiologies of cancer cachexia, resulting in weight loss due to degradation of skeletal muscle and adipose tissue, and suppression of appetite [14,37]. C-reactive protein (CRP) levels have been reported to be associated with weight loss, decreased QOL, and shorter survival in advanced cancer patients [38]. The Glasgow prognostic score (GPS), a scoring system that combines CRP and albumin levels, is a prognostic score for cancer patients that is independent of the stage of the disease [39]. The cut-off values for GPS are set at CRP 1.0 mg/dL and albumin 3.5 g/dL, and patients with CRP levels above 1.0 mg/dL and albumin levels below 3.5 g/dL are considered to have the poorest prognosis [39]. The usefulness of modified GPS (cut-off value of CRP = 0.5 mg/dL) has also been reported [40].

### 4.2. Mechanism for Cachexia

Proteolysis-inducing factor (PIF) secreted by cancer cells induces apoptosis via activation of caspases, inhibits protein synthesis in skeletal muscle, and accelerates proteolysis [41,42,43]. The lipid-mobilizing factor (LMF) secreted by cancer cells promotes lipolysis, converting triglycerides into fatty acids and replacing white adipocytes with brown adipocytes, which are more thermogenic [17]. Proinflammatory cytokines accelerate proteolysis via the ubiquitin proteasome pathway [44,45]. Inflammatory cytokines such as TNFα enhance glycogenesis in the liver via insulin resistance [46]. Furthermore, the consumption of glucose by cancer cells depletes glycogen in the liver, further increasing glycogenesis and promoting the degradation of fat and skeletal muscle [46]. On the other hand, parathyroid hormone-related protein (PTHrP) is produced in large amounts by tumor cells and causes hypercalcemia due to calcium reabsorption in the kidney and calcium mobilization from bone [4]. Hypercalcemia associated with malignancy is the most frequently observed tumor-associated syndrome, occurring in approximately 10% of patients with advanced cancers of lung, kidney, breast, head and neck, and bladder, and in 30% of patients with end-stage cancer [47,48]. A Spanish research group examined adipose tissue at various time points after cancer transplantation in mice that had been transplanted with cancer cells and suffered from cachexia and found that brown adipocytosis began at an early stage [49]. As mentioned earlier, brown adipocytes have a high heat-producing capacity, while white adipocytes are energy-storing cells [50]. They also found that a molecule called uncoupling protein 1 (UCP1) is expressed in adipose tissue and converts energy into heat in the mitochondria. In addition, inflammatory cytokines such as IL6 were elevated in mice with cachexia. In mice in which these inflammatory cytokines are suppressed, the expression of UCP1 molecules and brown adipocytosis was inhibited, and weight loss did not occur even when cancer progressed [49]. PTHrP stimulates brown adipocytes to produce UCP1 [51].

Adipose wasting is also an important symptom in cancer cachexia. In cancer cachexia, adipose can be lost faster than skeletal muscle. In cachexia patients, fat mobilization is first activated and adipose wasting occurs. Next, white adipocytes turn into beige adipocytes, which express genes characteristic of brown adipocytes, such as UCP1. Beige adipocytes function similarly to brown adipocytes in terms of energy expenditure, resulting in a negative energy balance [52]. TNF-α secreted by cancer cells can inhibit glucose transport and lipogenesis by decreasing the expression of glucose transporter 4 [53]. Hanayama et al. found that a protein called proliferin-1 secreted by cancer cells acts on adipocytes to regulate the inhibition of adipogenesis and the promotion of lipolysis [54]. They found that transplantation of cancer cells into normal mice results in adipose tissue loss and adipose wasting due to cachexia, but this loss is suppressed in the case of cancer cells lacking proliferin-1 [54]. Cancer-induced IL-6, leukemia inhibitory factor, etc. are also associated with adipose wasting [55,56,57].

### 4.3. Anorexia and Appetite-Related Hormones in Cancer Cachexia

**Figure 2:**
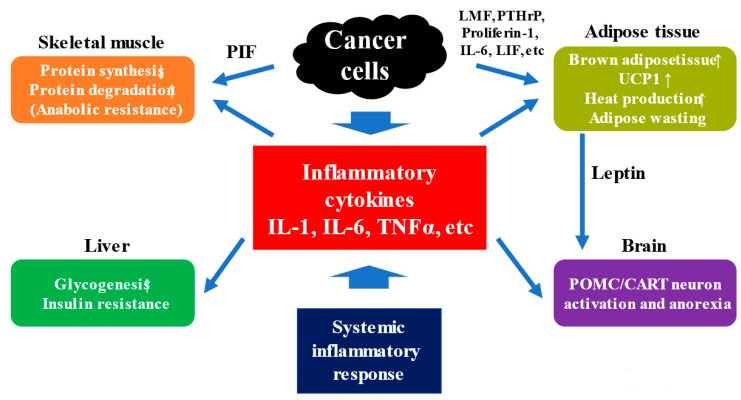
Mechanism for cancer cachexia. PIF; proteolysis-inducing factor, LMF; lipid-mobilizing factor, PTHrP; parathyroid hormone-related protein, LIF; leukemia inhibitory factor, UCP1; uncoupling protein 1, POMC; proopiomelanocortin, CART; cocaine and amphetamine-regulated transcript.

Anorexia is one of the main signs of cancer cachexia and is associated with weight loss. Recent studies have pointed out the involvement of the neuroendocrine system in cancer cachexia, and the role of the hypothalamus, pituitary gland, and adrenal gland, which are the control centers of appetite, has attracted attention. The main components are neuropeptide Y (NPY) [58] and agouti gene-related protein (AgRP) [59], which promote appetite, and proopiomelanocortin (POMC) [60] and cocaine and amphetamine-regulated transcrip (CART) [61], which suppress appetite. In cancer cachexia, chronic inflammation promotes the expression of proinflammatory cytokines in the hypothalamus, leading to inactivation of NPY/AgRP neurons and activation of POMC/CART neurons, resulting in various symptoms such as anorexia [6,7]. Anorexia can be enhanced by physical symptoms such as pain, fever, diarrhea, abdominal pain, and respiratory distress, as well as psychiatric symptoms such as depression and delirium [6,7]. On the other hand, recently, ghrelin, an endogenous hormone secreted by the stomach to promote appetite, has been attracting attention as a therapeutic target for anorexia associated with cancer cachexia [62]. Ghrelin not only promotes appetite, but also has anti-inflammatory effects, inhibits muscle protein degradation via MuRF-1/MAFbx, promotes muscle protein synthesis via IGF-1, inhibits apoptosis, increases fat storage, and decreases energy expenditure, and is thought to ameliorate multiple pathologies of cancer cachexia [63]. In a study of a small number of patients, natural ghlerin administration to patients with advanced cancer was reported to be well tolerated and improve nutritional status [64]. Leptin, secreted by adipose tissues, is an anti-obesity hormone that suppresses appetite by acting on leptin receptors in the hypothalamus [65]. Leptin receptors are increased in patients with cancer cachexia, and the appetite-suppressing effect is enhanced [65]. The relationship between cancer cachexia and skeletal muscle, adipose tissue, liver, and brain is shown in Figure 2.

## 5. Non-Pharmacological Therapies for Cancer Cachexia

### 5.1. Clinical Evidence for the Treatment of Cancer Cachexia

As refractory cachexia is difficult to treat, early diagnosis and early intervention are necessary [30,66]. In addition, as mentioned earlier, multiple factors are involved in cancer cachexia, including anorexia, skeletal muscle loss, and metabolic changes in the liver and adipose tissue against a background of systemic inflammation. Therefore, the treatment of cancer cachexia requires not only pharmacological therapies but also multidisciplinary interventions including nutrition therapy, exercise, and psychosocial interventions [26,67,68,69]. However, clinical trials of pharmacological therapies and exercise interventions in patients with cancer cachexia have reported high dropout rates and low compliance, and the treatment itself can be a burden for patients [70,71]. The aging of cancer patients and the diversification of cancer treatments further increase the physical and psychological burden on patients, making it difficult for them to continue treatment. Thus, the treatment of cancer cachexia should be chosen in a way that can be continued according to the patient’s condition and lifestyle [26]. There are not many randomized controlled trials (RCTs) of non-pharmacological treatments (i.e., nutrition and exercise) in patients with cancer cachexia [72,73]. In RCTs in patients with advanced cancer, the nutritional therapy alone has not demonstrated consistent efficacy on weight, survival, etc. [70,74]. Regarding exercise, patients with advanced cancer who can complete an exercise program show improvements in physical function and QOL [75,76,77,78,79,80,81,82,83], and the ESMO guidelines recommend resistance exercise 2–3 times per week if possible for patients with cachexia [26]. In Japan and abroad, clinical trials of multidisciplinary interventions combining nutritional and exercise therapies have been conducted, and their efficacy and safety have been verified [83,84,85,86,87,88]. The ESPEN guidelines and the ESMO guidelines state that nutritional care should always be accompanied by exercise training [26,32,89]. The ESMO guidelines also recommend regular nutritional assessment and nutritional intervention in patients with advanced cancer who have a prognosis of 3–6 months or longer, and less nutritional intervention in patients with a prognosis of less than 3–6 months [26]. Refractory cachexia is a stage that does not respond to nutritional administration, and it is reasonable to reduce the nutritional dosage toward the end of life, when metabolic abnormalities become more severe. Aggressive artificial nutrition does not alleviate cachexia in the terminal stage [32]. A significant increase in catabolism may cause a metabolic burden that can be harmful to the human body. Especially in intravenous nutrition, excessive infusion leads to edema and exacerbation of pleural fluid and ascites [32,89].

### 5.2. Energy Metabolism and Nutritional Support in Cancer Cachexia

Total energy expenditure (TEE) is the sum of REE and activity energy expenditure (AEE). REE is the sum of basal energy expenditure (BEE) and diet-induced thermogenesis (DIT). In other words, TEE = REE + AEE = BEE + DIT + AEE. It has been reported that TEE was increased in approximately half of the weight loss group of cancer patients [90] and that REE was increased in approximately half of the patients at the time of cancer diagnosis [91]. The REE of cancer patients, however, differs according to cancer type and cancer clinical stage [32]. In advanced cancer patients with refractory cachexia, REE is increased in cases with high inflammatory response, but TEE tends to decrease due to decreased daily activity (i.e., decreased AEE), so there is no need of high-energy administration for them. Therefore, the ESPEN guidelines recommend TEE to be set at 30–35 kcal/kg/day in outpatients and 20–25 kcal/kg/day in bedridden patients [32,89]. The use of the intestinal tract is an important route of nutrition for cancer patients. Many studies have shown that intestinal nutrition improves immunocompetence more than intravenous nutrition [92]. In both pre-cachexia and cachexia, if oral intake is possible, nutrient requirements should be supplemented orally as much as possible, for example, with oral nutritional support (ONS) [32,93]. The ESMO guidelines also recommend ONS [26]. When oral intake alone is insufficient, artificial nutrition and hydration (ANH) should be considered [32,92]. In cancer patients, glucose intolerance is often impaired but lipid metabolism is preserved, and thus fat administration is recommended [21]. On the other hand, it has been reported that intravenous administration of amino acids at 2 g/kg/day for highly malnourished cancer patients and 1.5 g/kg/day for moderately malnourished cancer patients improves cancer chemotherapeutic tolerability [94]. It has been reported that a high-protein dietary supplement enriched with leucine, one of the branched-chain amino acids, promotes protein synthesis in skeletal muscle of cancer patients [95]. Glucose intolerance is reduced in patients with advanced cancer. Therefore, infusion with high concentrations of carbohydrates may cause hyperglycemia [32].

## 6. Pharmacological Therapies for Cancer Cachexia

### 6.1. Anamorelin

Pharmacological therapies for cachexia have limited efficacy and are difficult to improve the severely reduced muscle mass in patients with cachexia. Corticosteroids [96], non-steroidal anti-inflammatory drugs [97], androgens [98], progestins [99], cannabinoids [100], and other drugs have been reported to be useful in patients with cachexia, but their efficacy was limited to improvement of QOL. However, with the advent of drugs such as ghrelin receptor agonists [15] and selective androgen receptor modulators [101], which have been shown to improve muscle mass in cachexia patients, future developments in pharmacotherapy are expected. Anamorelin, a ghrelin receptor agonist, is currently the only drug available for the indication of cancer cachexia in Japan [15,102]. In Europe and the USA, two overseas phase III trials (ROMANA1 and ROMANA2 [103]) were conducted. Co-primary endpoints (12-week mean change from baseline in lean body mass (LBM) and 12-week mean change from baseline in grip strength) were established. In both studies, 12-week mean change from baseline in LBM showed significance in the anamorelin group vs. the placebo group, but 12-week mean change from baseline in grip strength did not show significance in the anamorelin group vs. the placebo group. This is why anamorelin was not approved for the pharmaceutical use in Western countries. In the Japanese RCT of anamorelin, the primary endpoint, 12-week mean change in LBM from baseline, showed a statistically significant difference in the anamorelin 100 mg group (80 patients) vs. the placebo group (92 patients) (1.38 ± 0.18 kg vs. −0.17 ± 0.17 kg, *p* < 0.0001) [104]. Furthermore, in the Japanese single-arm study, the primary endpoint, the percentage of subjects who maintained or increased LBM, was 31 (63.3%) out of 49 subjects in the anamorelin 100 mg group, which was higher than the threshold response rate (30.7%) set in advance [105]. In addition, there was a trend toward improvement in appetite-related QOL in the anamorelin 100 mg group in both studies. Based on these results, anamorelin has been approved for the treatment of cancer cachexia in Japan. It has been reported that anamorelin elevates IGF-1, and IGF-1 promotes tumor growth [103]. However, compared to placebo, there was no clear trend of increased tumor progression due to anamorelin [15,103,104,105].

### 6.2. Enobasarm, MABp1, and Others

Enobosarm is a selective androgen receptor modulator [106]. In a phase II trial of enobosarm, patients with advanced cancer who had lost at least 2% of their body weight in the last 6 months were given enobosarm 1 mg, enobosarm 3 mg, or placebo orally once daily. There was a significant increase in LBM in the enobosarm 1 mg group and the enobosarm 3 mg group compared to baseline, but no significant change was found in the placebo group [106]. Subsequently, the phase III trial of enobosarm was conducted, but the results of the trial have not been published. Cytokines such as IL-1α and IL-6 cause fever, malaise, anorexia, and skeletal muscle loss. MABp1, a monoclonal antibody, specifically binds to IL-1α and attenuates its action [107]. A phase III comparative study of MABp1 was conducted in 333 patients with unresectable or metastatic colorectal cancer with cancer cachexia. The results showed a significant improvement in cachexia symptoms and stability of LBM in the MABp1 group compared to the placebo group [107]. However, the European Medicines Agency (EMA) rejected the application on the grounds that (1) there was no clear improvement in LBM and QOL by MABp1, and (2) although incidence of serious adverse event (SAE) was 23% in the MABp1 group and 32% in the placebo group, there was an unacceptable increased risk of infection of MABp1 in vulnerable patients receiving palliative care. Recombinant human growth hormone (GH) may be promising for muscle mass loss [108]. The GH/IGF-1 signaling is a main anabolic pathway in the skeletal muscle. In a previous RCT, patients with HIV-associated muscle wasting treated by recombinant human GH showed an increase in LBM and weight in a dose-dependent manner [108]. In cancer cachexia, increased gut permeability can promote the diffusion of proinflammatory or inflammatory molecules on the intestinal barrier, resulting in systemic inflammation [109]. Thus, pharmacological intervention for dysbiosis in cancer cachexia may be beneficial, however there is no sufficient clinical evidence regarding this. The usefulness of anti-inflammatory cytokine therapies such as anti-TNFα therapy, anti-IL6 therapy, and anti-IL1α therapy on cancer cachexia have been reported [57]. The usefulness of myostatin inhibitors on cancer cachexia have also been reported [57]. However, these drugs have not received the approval of use by the regulatory agencies.

## 7. Multidisciplinary Intervention Model

**Figure 3:**
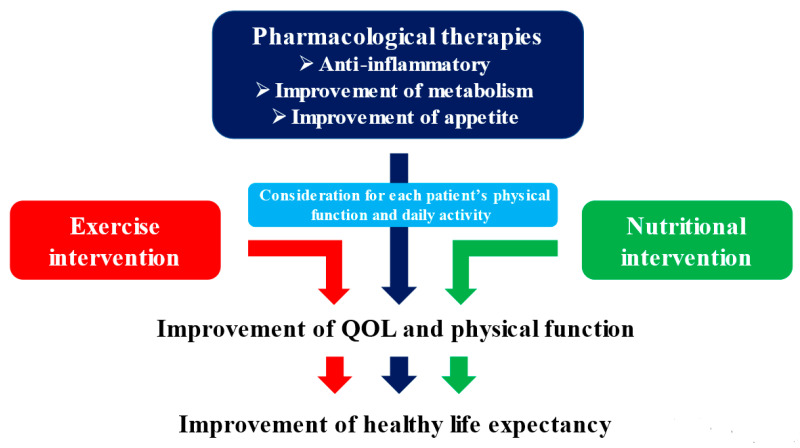
Multidisciplinary intervention for cancer cachexia.

As we have discussed, cancer cachexia is a complex metabolic disorder that is difficult to improve with conventional nutritional therapy, and nutritional support that takes into account its pathophysiology is important. According to the ESPEN and ESMO guidelines, the treatment of cancer cachexia requires a combination of nutrition, exercise, and pharmacotherapy [26,32,89], and the evidence level for nutritional counseling is also high [26,110,111]. A multidisciplinary intervention model for cancer cachexia is presented in Figure 3. In addition to anti-inflammatory, metabolism-improving, and appetite-improving medications, high-quality nutritional therapy and appropriate exercise tailored to the patient’s physical function can help improve physical function as well as increase skeletal muscle mass [112,113,114]. These strategies will become even more necessary in the future.

## 8. Final Remarks

This review outlined (1) definition, (2) mechanisms, and (3) treatment in patients with cancer cachexia. The diagnosis of cachexia is never easy, especially in the diagnosis of pre-cachexia, and clinicians should be aware of this possibility from an early clinical stage of cancer patients. In patients with advanced cancer, various metabolic abnormalities occur due to substances secreted by cancer cells and immunological responses. Multidisciplinary treatment for cancer cachexia is the mainstay of cachexia treatment, and multidisciplinary collaboration centered on nutritional support is important.

## Figures and Tables

**Table 1:** Cachexia and starvation.

	Cachexia	Starvation
**Body weight**	**↓**	**↓**
**Skeletal muscle**	**↓**	**→**
**Adipose tissue**	**↓**	**↓**
**Rest energy expenditure**	**↑**	**↓**
**Inflammatory protein**	**↑**	**→**

## Data Availability

Data sharing not applicable.

## Abbreviations

REE	resting energy expenditure
BMI	body mass index
ESPEN	European Society of Clinical Nutrition and Metabolism
CSS	cachexia staging score
ESMO	European Society of Medical Oncology
CRP	C-reactive protein
GPS	Glasgow prognostic score
PTHrP	parathyroid hormone-related protein
UCP1	uncoupling protein 1
NPY	neuropeptide Y
AgRP	agouti gene-related protein
POMC	proopiomelanocortin
CART	cocaine and amphetamine-regulated transcript
RCTs	randomized controlled trials
TEE	total energy expenditure
AEE	activity energy expenditure
BEE	basal energy expenditure
DIE	diet-induced thermogenesis
ONS	oral nutritional support
LBM	lean body mass
GH	growth hormone
